# The Sm29 antigen differentially shapes transcriptomic and regulatory landscapes across reactional forms of leprosy

**DOI:** 10.3389/fimmu.2026.1844112

**Published:** 2026-06-01

**Authors:** Joyce Karoline Silva, Lucas Neves de Farias, Tainã Lago, Luciana dos Santos Cardoso, Ricardo Khouri, Paulo Roberto Machado, Léa Cristina Castellucci

**Affiliations:** 1 Programa de Pós-graduação em Ciências da Saúde da Universidade Federal da Bahia, Salvador, Brazil; 2 Laboratório de Medicina e Saúde Pública de Precisão – MESP² – Instituto Gonçalo Moniz-FIOCRUZ, Bahia, Brazil; 3 Serviço de Imunologia da Universidade Federal da Bahia, Salvador, Brazil; 4 Instituto Nacional de Ciência e Tecnologia em Doenças Tropicais, Salvador, Brazil; 5 Instituto Nacional de Ciência e Tecnologia em Saúde Digital (INCT-DigiSaúde), Salvador, Brazil; 6 Department of Microbiology, Immunology and Transplantation, Rega Institute for Medical Research, Clinical and Epidemiological Virology, Leuven, Belgium

**Keywords:** immune response, leprosy, regulation, Sm29 antigen, transcriptome

## Abstract

**Background:**

It is documented that *Schistosoma mansoni* antigens can induce immune responses able to regulate complex diseases. Conversely, the dysregulated inflammatory response in leprosy increases morbidity and leads to reactional episodes, impairing the disease pathogenesis. The goal of this study was to evaluate the potential of the *S. mansoni* (Sm29) antigen in regulating the immune response in leprosy through a transcriptome study across clinical reactional forms: reversal reaction (RR), and erythema nodosum leprosum (ENL), and without reaction (WR).

**Methods:**

Peripheral blood mononuclear cells (PBMCs) were cultured and stimulated with sonicated *M. leprae* antigen and rSm29 antigen. Total RNA was extracted by the TRIzol^®^ (Ambion) according to the manufacturer’s protocol. RNA libraries were constructed with the TruSeq Stranded Total RNA Prep Globin kit (Illumina) and quantified by qPCR with the KAPA Library Quantification Kit (Roche). Sequencing was performed by Nextseq 2000 (Illumina) with 2x100 base pairs (bp) reads. Differential expression was analyzed using DESeq2 to identify differentially expressed genes (DEGs) between conditions. Identified DEGS were subjected to Ingenuity Pathway Analysis (IPA)for enrichment pathway analysis.

**Results:**

The Sm29 antigen was able to modulate immune response pathways in leprosy, with this response being more pronounced in the reactional forms of the disease, especially in RR. Interestingly, when compared to sonicated *M. leprae*, Sm29 had an opposite effect in RR-ENL, evidenced by the top genes most expressed in RR and less in the ENL (*C2CD4B, CEMIP, IL23A, CD7, GPC5, EBF1, SIGLEC15, ADAT2, ZC3H12D*); Additionally, IPA analysis shown that in RR, modulation of the *CSF2* by Sm29 increases *IL10*, while modulation by IFN-α modulates *CXCL9, CXCL-10, IL15, TLRs 2* and *4*; On the other hand, in ENL, modulation of *IL1B* and *FN1* genes by rSm29 appear related to several chemokines and cytokines such as *IL6, CCL2* and *CXCL8*.

**Conclusion:**

Leprosy proves to be an efficient model for investigating products capable of regulating the host inflammatory response. Here, we open perspectives for new insights in the use of immunobiological, expanding attention to this field that might help management of leprosy patients, especially in reactional episodes.

## 1 Introduction

Leprosy is a neglected infectious disease caused by the bacilli *Mycobacterium leprae* and as a secondary causative agent, *Mycobacterium lepromatosis*. The disease primarily affects the skin, then progressing to a secondary stage, causing peripheral neuropathy with potential long-term disability (1). In 1966, Ridley and Jopling created a classification of leprosy based on variations in the patients’ immune response that range from self-cure to five spectral clinical forms: tuberculoid, borderline-tuberculoid, borderline-borderline, borderline-lepromatous, lepromatous (2). In addition, approximately of 30-40% of leprosy patients are diagnosed with sudden acute inflammatory reactions: type 1 reactions or RR are mediated by exacerbated cellular immunity against *M. leprae* antigens and present clinically as neuritis and/or inflammation and erythema of skin lesions; type 2 or ENL form, comprise activation of intermediate monocytes and other immune cells, immune complexes in several tissues, and high *TNF* production, with the abrupt appearance of subcutaneous nodules, in addition to systemic clinical manifestations such as fever, arthritis, myalgia, neuritis, and others which may require hospitalization for control (3, 4). The use of corticosteroids for treatment of RR and Thalidomide for the treatment of ENL is recommended by the World Health Organization (WHO), however these are therapeutic resources with recognized side effects and increased morbidity (5).

*Schistosoma mansoni* infection or associated products can down-modulate the type 1 CD4^+^ T cell inflammatory response characteristic of autoimmune diseases (6). The Sm29 is a membrane-bound glycoprotein located on the tegument of the adult worm and lung stage schistosomula (7). The recombinant Sm29 protein (rSm29) has been shown to be a molecule with immunomodulatory capacity in infectious inflammatory diseases such as myelopathy (HAM/TSP) caused by the Human T-cell Lymphotropic Virus (HTLV-1) virus and cutaneous leishmaniasis (CL) caused by *Leishmania braziliensis*. Preliminary data demonstrated that recombinant Sm29 reduced the production of *IFN-γ* and *TNF* in mononuclear cells from more than 60% of patients with CL *in vitro* (6, 8) thus identifying the immunomodulatory potential of Sm29 in regulating a Th1-type immune response. Following the same line, recombinant Sm29 was also tested in cell cultures from patients with HTLV-I. When rSm29 was added to PBMC cultures, there was a reduction in the production of *IFN-γ* by cells from 50% of these patients (9). In both diseases, the immunomodulatory effect was related to an increase of the *IL-10* levels, induced by Sm29.

On the other hand, evidence has been accumulating demonstrating that chronic infection with helminths, particularly *Schistosoma sp* or parasite products, can modulate the Th2-type inflammatory response in allergic-based diseases, such as asthma (10, 11). Recently, in a clinical trial performed with CL patients, a nanoformulation of Sm29 was used topically as an adjuvant along with Meglumine Antimoniate (MA) to treat the disease. The use of MA + rSm29 leads to a superior healing rate (71%) against MA alone (43%) as well as significant shorter healing time of lesions in the subjects that received MA + rSm29 (12).

There is no data regarding the effects of the Sm29 antigen on cells from leprosy patients. This pioneering study delves into the transcriptomic profile of PBMCs from leprosy patients, including those experiencing leprosy reactions, through RNA-sequencing analysis following stimulation with recombinant Sm29 antigen. By leveraging high-throughput sequencing, the research aims to uncover the molecular mechanisms underlying the immune response modulated by rSm29 in leprosy.

## 2 Materials and methods

### 2.1 Patients and clinical data

Twelve patients, four WR, four with RR and four with ENL were selected based on their diagnosis, following the Ridley-Jopling criteria, after clinical dermato-neurological evaluation and data from histopathology and bacillary index. The age mean was 45.89 ± 16.30 and samples were collected from March 2022 to March 2023 in the reference centers for treatment of leprosy at Hospital Universitario Edgard Santos and the Couto Maia Institute, both located in Salvador, Bahia, Brazil. For the WR group, we selected patients at the initial diagnosis, before starting MDT. However, in the case of RR and ENL, as they normally were undergoing treatment when developed reactions, we only selected those who arrived at the clinics without using immunosuppressants such as prednisone or thalidomide to avoid the bias of recruiting subjects under immunosuppressive treatment. Details in **Table 1**.

**Table 1:** Clinical-epidemiological characteristics of participants.

Patient data	NON-REACTIONAL	REACTIONAL
	(n=4)	(n=8)
Age (Mean ± SD*)	52,50 ± 19,74	41,13 ± 13,62
Gender, %		
Male	3/4 (75%)	5/8 (62,5%)
Female	1/4 (25%)	3/8 (37,5%)
Clinical phenotype		
Paucibacillary (PB), %		
Tuberculoid Leprosy (TL)	0/0	0/0
*Borderline-Tuberculoid* (BT)	0/0	3/8 (37,5) (RR*)
Indeterminate (I)	1/4 (25%)	0/0
Multibacillary (MB), %		
*Borderline Borderline* (BB)	1/4 (25%)	1/8 (12,5%) (ENL*)
*Borderline-Lepromatous* (BL)	0/0	1/8 (12,5%) (RR*)
		1/8 (12,5%) (ENL*)
Lepromatous leprosy (LL)	2/4 (50%)	2/8 (25%) (ENL*)
Patients in reactional states, %		4 (RR*)
		4 (ENL*)
Bacterial Index (Median, IR*)	3,9 (0,2-3,9)	0,3 (0-2,6)

RR*, Reversal Reaction; ENL*, Erythema Nodosum Leprosum; SD, Standard Deviation; IR*, Interquartile Range.

### 2.2 Cell culture

PBMCs were obtained from 30mL heparinized blood, using Ficoll Hypaque™ Plus density gradiente (GE healthcare) and adjusted to a concentration of 3 x 10^6^ cells/ml in RPMI 1640 (Life technologies) with 10% inactivated AB+ human serum, gentamicin (100 U/ml), L-glutamine (2mM) and HEPES (25mM). Cultures were stimulated with 1 – Medium; 2 – *M. leprae* sonicated antigen (ML) (20 µg/mL), strain NR−19329, provided by bei resources (beiresources.org); 3 – rSm29 (10 µg/mL) and cultured at 37 °C and 5% CO2 in propylene tubes for 12 and 18 hours. In cell cultures where rSm29 was present, 15µl of polymyxin B (diluted from stock at 1mg/mL) was added during incubation to eliminate possible effects of LPS. Cells were stored with TRIzol and kept at -70 °C until use.

### 2.3 ELISA chemokine assays

Levels of the cytokine and chemokines: *TNF*, *IL-6*, *IL-10*, *IL-8, IFNG, IL-4, IL-5, CXCL-9, CXCL-10* and *MCP-1* were measured in supernatants using commercial kits from R&D (R&D systems Inc. Minneapolis, MN, US) and BD OptEIA™ Set human (BD Biosciences, San Jose, CA, US), respectively, according to manufacturer’s protocols. Optical density was measured in the spectrophotometer at 450 nm. The results were expressed in pg/mL. Statistical analysis was performed using the Kruskal-Wallis test, with significance set at p ≤ 0.05.

### 2.4 RNA preparation and treatment

The PBMCs RNA was obtained using the miRNeasy^®^ Mini Kit (Qiagen) as follows: a) samples were first exposed to erythrocyte lysis using Buffer EL (Quiagen); b) afterwards, cells were disrupted in 700 μl of Qiazol Lysis Reagent with vortex homogenization for 1 minute, followed by 5 minutes incubation (RT); c) 140 μl of chloroform was added to each tube and material was centrifuged at 12000g for 15 minutes at 4°C; d) the aqueous phase formed, containing the RNA, was carefully aspirated and transferred to a collection tube (1.5 ml) and 525 μl of 100% ethanol was added to each tube; e) after homogenization, 700 μl of the sample was pipetted onto a column; f) The tubes were centrifuged and the supernatant was discarded; g) The RNA was resuspended in 25μl of RNase-free water and concentration determined by optical density using Nanodrop^®^. RNA was then treated with dsDNase (ThermoScientific) following purification, according to manufacturer’s instructions; h) RNA quantity and integrity were determined using a Qubit^®^ 2.0 Fluorometer (Thermo Fisher Scientific, Coon Rapids, Minnesota, USA) with the Qubit RNA high-sensitive assay kit).

### 2.5 RNA library construction and sequencing

Libraries were constructed from 200 ng of total RNA with the TruSeq Stranded Total RNA Prep Globin kit (# cat 20020612, Illumina) according to the manufacturer’s manual. After assembling the libraries, they are quantified by qPCR with the KAPA Library Quantification Kit (Roche) to estimate the concentration of each one in nM (nanomolar). From equimolar amounts of each library, a pool was prepared and sequenced on Nextseq 2000 (Illumina) with paired end 2x100 bp reads. Raw sequencing data generated in this study have been deposited in the NCBI Sequence Read Archive (SRA) under BioProject accession number PRJNA1463881.

### 2.6 Data processing and computational analysis

Quality control of raw sequence data was conducted using FastQC v0.11.8 and RSeQC v5.0.0 (13), with results summarized using MultiQC v1.13 (14). Low-quality sequences and adapter contamination were removed using Trimmomatic v0.39 (15).The trimmed reads were then aligned to the human reference genome GRCh38 (release 43, GENCODE) using the STAR v2.7.10a aligner (16). Exon-level read counts were generated using featureCounts v2.0.1 (17) utilizing genome annotation data. For differential expression analysis, DESeq2 v3.19 (18) was applied. Wald tests were used to assess gene-level differences, with contrasts defined for each clinical form across the three experimental conditions. Log2 fold-change estimates were refined using the apeglm shrinkage method to improve stability of effect size estimation. DEGs were defined using an adjusted p-value (FDR) threshold of ≤ 0.05, combined with a fold-change cutoff of ≥ 1 for upregulated genes and ≤ -1 for downregulated genes.

For the functional enrichment analysis, we employed the IPA software (QIAGEN Inc., version 2024), a specialized platform that integrates manually curated biological knowledge. The previously identified DEGs, along with their corresponding fold-change values and statistical significance, were uploaded to IPA for comprehensive analysis. Within IPA, we applied Disease and Biological Functions (DB) and Canonical Pathways (CP) modules were used to identify significantly enriched metabolic, signaling, and regulatory pathways present in the dataset, leveraging IPA’s extensive curated database. Additionally, the Upstream Regulators (UTR) module was applied to predict regulatory molecules that may be activated or inhibited, based on the expression patterns of their known downstream targets. Complementing these analyses, the Regulator Effects tool was used to build integrated three-layer networks connecting upstream regulators, target genes and biological outcomes.

### 2.7 Deconvolution

Gene expression data normalized via DESeq2 was used as input for CIBERSORTx (19) with the LM22 as signature matrix. Relative immune cell fractions were estimated, ranging from 0 to 1, representing the proportion of each population within the sample. For RNA−Seq data, quantile normalization was disabled, as recommended by the developers to preserve distributional properties. Statistical analysis was performed using the Kruskal-Wallis test, with significance set at p ≤ 0.05.

### 2.8 Ethics statement

The study has been approved by the Ethical Committee of the Hospital Universitário Prof. Edgard Santos, Federal University of Bahia, (CAAE: 79268017.0.0000.0049). All patients read and signed an informed consent form.

## 3 Results

### 3.1 Cytokine and chemokine profiles in leprosy patients according to reaction status and stimulation

**Figure 1:**
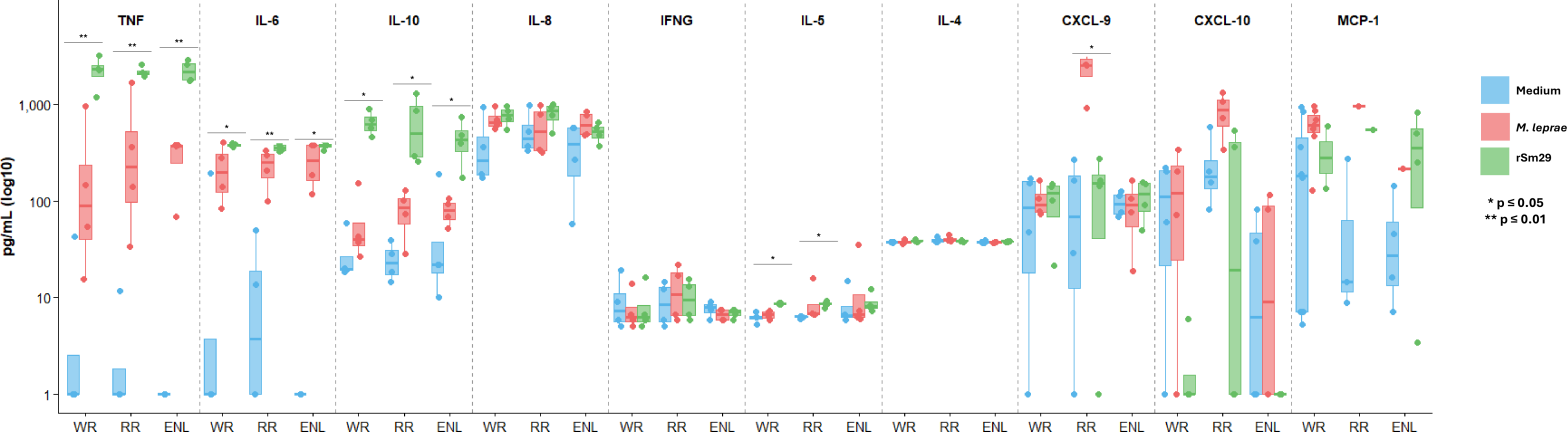
Stimulus−Specific Cytokine and Chemokine Responses in Leprosy: ENL, RR, and WR. Boxplots depict the distribution of cytokine and chemokine concentrations (pg/mL) for each analyte, stratified by clinical form (WR, RR, ENL), in each boxplot, the central line indicates the median; the box limits correspond to the first and third quartiles; the points represent individual samples. Within each clinical group, three stimulation conditions are shown: unstimulated medium (blue box; dark−blue points), *M. leprae* (pink box; dark−pink points), and rSm29 (green box; dark−green points). Statistical comparisons across the three clinical groups were performed using the Kruskal–Wallis test; asterisks indicate significance levels: p ≤ 0.05 (*), p ≤ 0.01 (**).

Cytokine and chemokine profiles in leprosy patients according to the presence or absence of reactions. Using the ELISA technique, we looked at circulating levels of cytokines and chemokines in serum of leprosy patients with reactions (ENL and RR, thereby called REAC) and without reactions (WR) under the three stimulation conditions of cell culture: 1- Medium; 2- *M. leprae*; 3 - rSm29 (**Figure 1**).

Significant differences were observed for *TNF*, *IL-6*, and *IL-10*, with *TNF* showing highly significant elevations in REAC and WR groups (p ≤ 0.01) under antigenic stimulation. *IL-6* and *IL-10* also demonstrated significant increases (p ≤ 0.05), with *IL-6* in the RR group reaching a stronger threshold (p ≤ 0.01), Notably, *IL-6* levels were markedly elevated under *M. leprae* and rSm29 stimulation compared to the unstimulated medium, suggesting a heightened antigen-driven inflammatory response characteristic of reversal reactions. In contrast, *IL-8* and *IFNG* did not exhibit notable differences between groups or stimulation conditions, indicating a relatively stable expression profile under the tested conditions. *IL-5* displayed a distinct expression pattern, with significantly elevated levels observed in both RR and WR patient groups (p ≤ 0.05). Notably, WR patients stimulated with rSm29 showed the highest *IL-5* concentrations, suggesting an enhanced antigen-specific response. *CXCL-9* levels were significantly elevated in RR patients compared to the other clinical groups (p ≤ 0.05), particularly under *M. leprae* stimulation, where concentrations were notably higher. This pattern suggests a reaction-specific chemokine response associated with reversal reactions. Despite not reaching statistical differences, the Sm29 antigen reduced the *CXCL-10* levels in all groups. No other cytokines or chemokines showed statistically significant differences across clinical groups or stimulation conditions.

### 3.2 Comparative transcriptomics of leprosy patients under rSm29 stimulus

To investigate the transcriptomic signature of different clinical forms, we employed Principal Component Analysis (PCA) on gene expression of PBMCs stimulated under three conditions: medium, *M. leprae*, and rSm29 antigen. This analysis focused on three distinct clinical groups — WR, RR, and ENL — to identify the major sources of variance and assess transcriptional responses across conditions.

**Figure 2:**
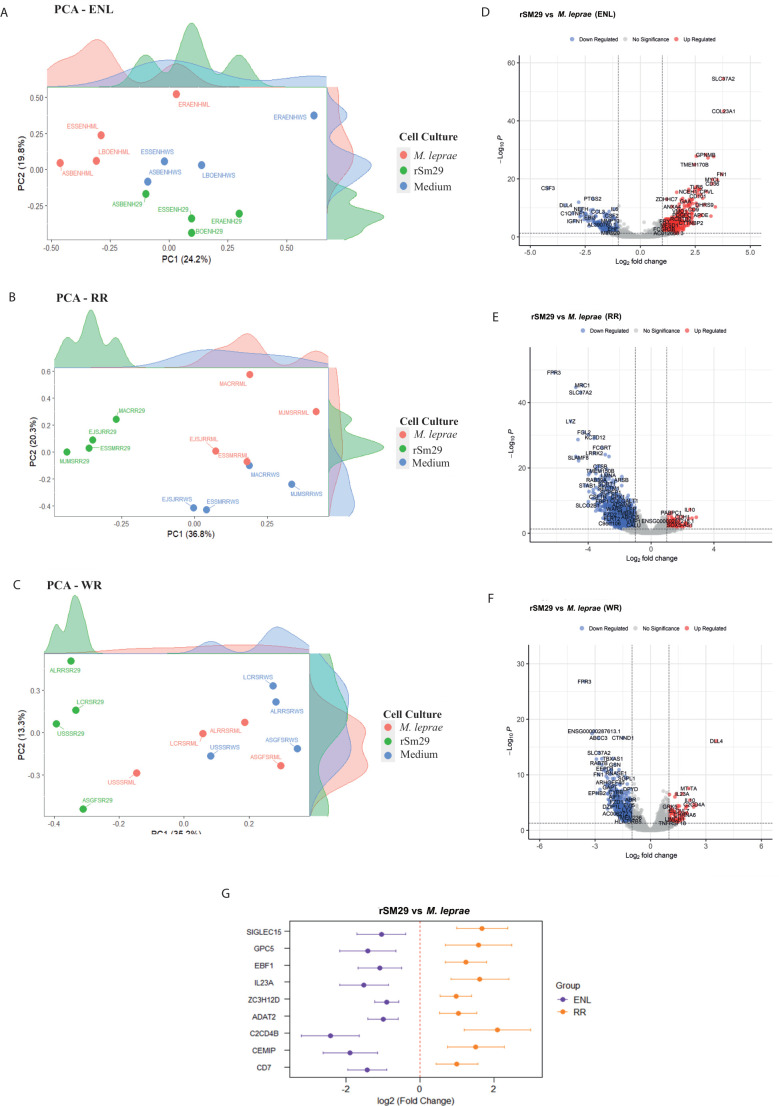
PCA of PBMC from clinical forms of leprosy under stimulation with medium, *M. leprae*, and rSm29, and distribution of DEGs in comparisons. **(A–C)** PCA of samples from the ENL, RR, and WR groups, respectively, stimulated with *M. leprae* (pink), rSm29 (green), and unstimulated medium (blue), illustrating gene dispersion along PC1 (top) and PC2 (right). **(D–F)** Volcano plots comparing rSm29 vs. *M. leprae* within each clinical form. DEGs are highlighted in red (up-regulated) and blue (down-regulated), while gray represents genes that did not meet the cutoff thresholds. The y-axis corresponds to p-value (-log10), and the x-axis to fold change (log_2_), with dotted lines marking statistical cutoffs. **(G)** Forest plot of differentially expressed genes in the ENL (purple) and RR (yellow) groups in rSm29 vs *M. leprae* comparison. The central point represents the log_2_ fold change, while the horizontal bars indicate the standard error.

In the WR group, PC1 accounted for 35.2% of the total variance, with PC2 capturing 13.3%. For the RR group, PC1 explained 31.8% of the variance, and PC2 contributed 20.3%. In the ENL group, PC1 represented 24.2% of the variance, while PC2 explained 19.8%. Notably, in both WR and RR groups, PC1 clearly separated samples stimulated with rSm29 from those exposed to WS or *M. leprae*, indicating a distinct transcriptional response induced by this antigen. In the ENL group, separation was more subtle, occurring predominantly along PC2. Supporting these findings, histograms further illustrate the distribution and separation of samples within the PCA space for each clinical group, emphasizing the differential impact of rSm29 stimulation on gene expression profiles. (**Figures 2A–C**).

To identify the DEGs, each stimulus was compared against the others within each clinical form. All groups showed DEGs, except for the WR group, where no DEGs were detected between the *M. leprae* and medium conditions — underscoring the specificity of the rSm29 response. Notably, the comparison between rSm29 and *M. leprae* stimulation revealed key transcriptomic differences across groups (**Figures 2D–F**). In the RR group, we identified 230 upregulated and 1,352 downregulated DEGs; in the ENL group, 496 upregulated and 270 downregulated DEGs; and in the WR group, 82 upregulated and 585 downregulated DEGs. According to fold change analysis, the top genes most highly expressed under rSm29 stimulation in the RR group — including *C2CD4B, CEMIP, IL23A, CD7, GPC5, EBF1, SIGLEC15, ADAT2* and *ZC3H12D* — were, conversely, among the least expressed in the ENL group in the same comparison (**Figure 2G**; **Supplementary Table 1**). These findings highlight distinct regulatory patterns between RR and ENL in response to rSm29, suggesting divergent immune activation pathways modulated by this antigen.

### 3.3 Distinct transcriptomic and pathway regulation profiles across clinical forms under rSm29 and other stimuli

**Figure 3:**
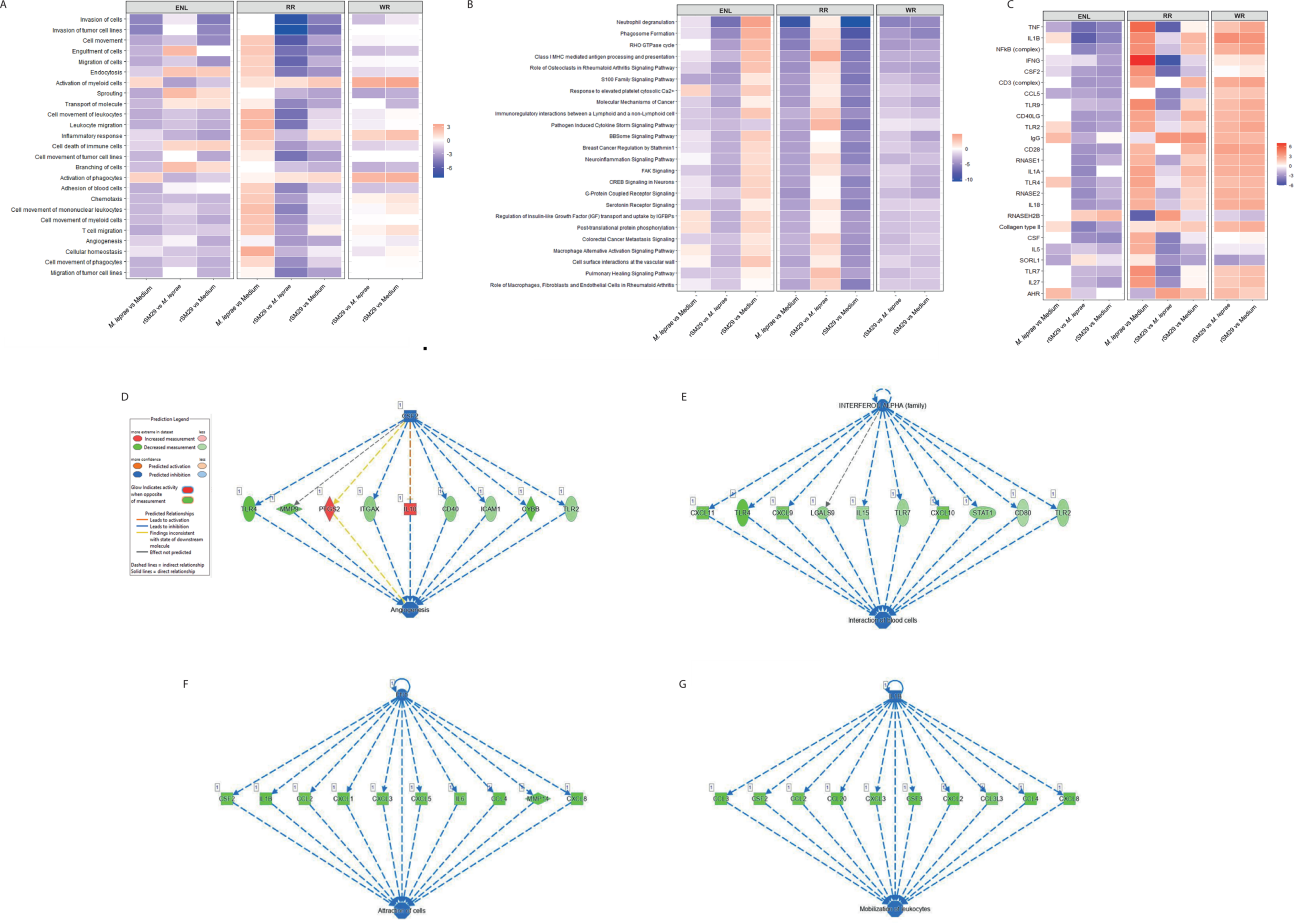
Enrichment analysis of diseases, biological functions, pathways, and upstream regulators and regulator effect networks based on IPA. Heatmaps display enrichment results in z-score, reflecting predicted activation or inhibition based on gene expression patterns. Orange blocks indicate activation, blue blocks indicate inhibition, and white blocks represent non-significant z-scores prediction within each comparison. **(A)** Heatmap representing the enrichment of diseases and biological functions. **(B)** Heatmap showing enriched canonical pathways. **(C)** Heatmap of UTR. In the Regulator Effects, at the top are the UTR influence gene expression, with autoregulation indicated by self-connected arrows. DEGs in the center are colored based on their predicted role: red for activated and green for inhibited. At the bottom, biological functions illustrate the impact of transcriptomic alterations. Dashed lines indicate regulatory influences, with orange arrows for activation and blue arrows for inhibition. **(C, D)** The RR group in the rSm29 vs. *M. leprae* comparison. **(E, F)** The ENL group in the rSm29 vs. *M. leprae* comparison.

The DEGs identified in the stimulus comparisons were analyzed using IPA to uncover DB relevant to the clinical forms of leprosy under distinct stimulation conditions. In both the RR and WR groups, rSm29 stimulation induced similar patterns of negative regulation in processes such as cell movement, cell migration, cell invasion and engulfment, endocytosis, phagocytosis, and the organization of the cytoskeleton and cytoplasm (**Figure 3A**). Notably, the RR group exhibited the most pronounced downregulation, highlighting the distinct immunomodulatory effect of rSm29 in this clinical form. In contrast, in the ENL group, the rSm29 versus *M. leprae* comparison showed the opposite pattern — consistent with the top DEG expression patterns comparison with RR — marked by the activation of these biological functions, whereas the other ENL comparisons followed the same regulatory trends observed in the RR and WR groups.

Similarly, the CP analysis revealed aligned regulatory patterns under rSm29 stimulation across clinical forms. In both RR and WR groups, rSm29 led to the negative regulation of key immune and inflammatory pathways, including neutrophil degranulation, phagosome formation, Class I MHC-mediated antigen processing and presentation, and neuroinflammation signaling (**Figure 3B**). Again, the RR group displayed the most pronounced downregulation across these pathways, reinforcing the unique immunomodulatory signature of rSm29 in this group.

Interestingly, the UTR analysis helped identify key molecular drivers underlying these transcriptional changes, highlighting molecules well-known in leprosy pathogenesis, such as *IL1B, TNF, IFNG, NFKB*, and *IL4* (**Figure 3C**). The convergence of upstream signals was particularly notable between REAC under rSm29 stimulation, contrasting with the patterns observed in WR. Once again, the rSm29 versus *M. leprae* comparison in RR displayed the strongest regulatory signal, underscoring that, although RR and WR share similar downstream biological functions under rSm29, the upstream activation patterns driving these effects diverge sharply between clinical forms.

Together, these results suggest that rSm29 exerts a distinct immunomodulatory effect by selectively dampening or activating immune pathways depending on the clinical context, with RR showing the most pronounced shifts both at the functional and regulatory levels. This highlights the importance of specific responses in shaping the immune landscape across diverse clinical presentations of leprosy.

### 3.4 Distinct regulatory networks induced by rSm29 across reactional forms

Among the networks generated through IPA analysis, two stood out in the RR group due to the strength of their predicted interactions and biological relevance, derived from the comparison between rSm29 and *M. leprae* stimulation. The first network identified *CSF2* as a central upstream regulator, predicted to negatively modulate key innate immune receptors (*TLR2*, *TLR4*) and adhesion molecules (*ICAM1*), while also promoting the expression of *IL-10* and *PTGS2*. This network is associated with angiogenesis with negative functions (**Figure 3D**).

In contrast, the second major network positioned *IFN-α* as a central upstream regulator, orchestrating a pro-inflammatory transcriptional negative response. This network included pattern recognition receptors (*TLR2*, *TLR4*) and key immune mediators such as *CXCL9*, *CXCL10* and *IL15*, all previously associated with the negative function of interaction of blood cells in RR (**Figure 3E**).

### 3.5 *FN1* and *IL1B* modulate *IL6* and chemokines through rSm29 in the ENL

To investigate the regulatory landscape driven by rSm29 in ENL patients, we performed an IPA focusing on the construction of Regulator Effects Networks. This strategy enabled the identification of potential causal relationships and biological mechanisms underlying the rSm29-induced transcriptomic response.

In the comparison between rSm29 and *M. leprae*, two negatively regulated networks were identified. In the first, *FN1* was predicted as an upstream regulator, with a downstream cascade involving multiple pro-inflammatory chemokines such as *CCL2, CCL4, CXCL1, CXCL3, CXCL5, CXCL8*, as well as cytokines *IL1B* and *IL-6*. This network was associated with the biological function of cell attraction, which was found to be negatively regulated (**Figure 3F**). The second network highlighted *IL1*{ιτ}B{/ιτ} itself as a negatively regulated upstream regulator, modulating a similar set of chemokines and linked to the mobilization of leukocytes, also under negative regulation (**Figure 3G**).

These molecules are well known for their roles in neutrophil recruitment and amplification of acute inflammatory responses — key drivers of ENL pathology. Interestingly, the suppression of these networks suggests that rSm29 dampens inflammatory signaling in the ENL context, potentially acting to restrain excessive immune activation. This contrasts with the inflammatory exacerbation typically seen in ENL and highlights the context-dependent immunomodulatory role of rSm29, which may shift between pro- and anti-inflammatory effects depending on the clinical form of leprosy.

### 3.6 Modulation by rSm29 has a similar profile between RR and WR

To broaden our understanding of the biological processes involved in the different clinical forms of leprosy under distinct antigenic stimuli, we first identified significantly modulated genes (FDR ≤ 0.05), regardless of fold change. By comparing these gene sets, we identified a shared transcriptional signature of 235 genes between RR and WR under rSm29 stimulation. Interestingly, these same genes were also modulated in ENL, particularly in the rSm29 vs *M. leprae* and *M. leprae* vs medium comparisons (**Supplementary Figure 2**).

**Figure 4:**
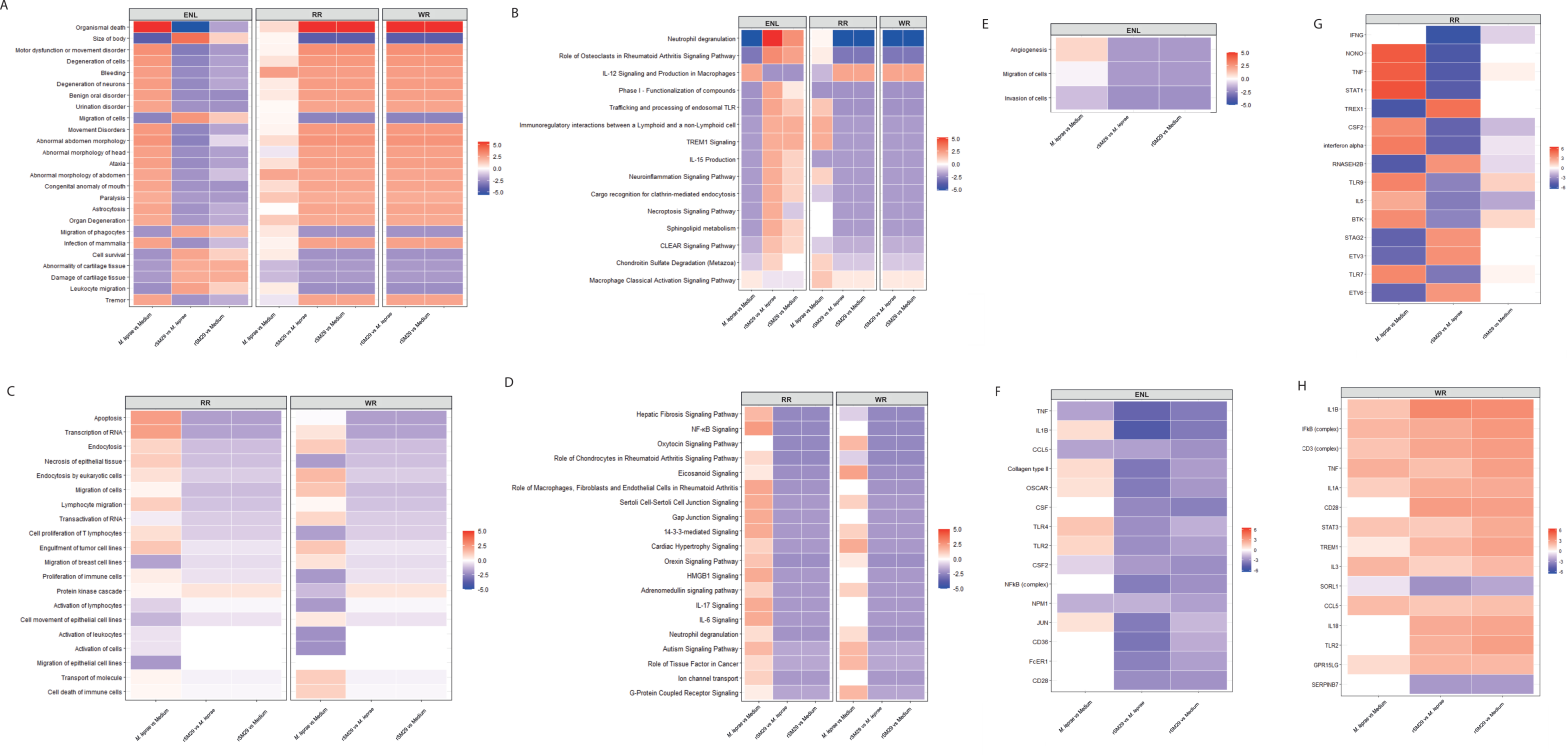
Enrichment analysis of diseases, biological functions, pathways, and upstreams of genes subsets. Heatmaps display enrichment results in z-score, reflecting predicted activation or inhibition based on gene expression patterns. Orange blocks indicate activation, blue blocks indicate inhibition, and white blocks represent non-significant findings within each comparison. **(A)** Heatmap representing the enrichment of diseases and biological functions in 235 genes subset. **(B)** Heatmap showing enriched canonical pathways in 235 genes subset. **(C)** Heatmap representing the enrichment of diseases and biological functions in 143 RR/WR genes subset. **(D)** Heatmap showing enriched canonical pathways in 143 RR/WR genes subset. **(E)** Heatmap representing the enrichment of diseases and biological functions in 23 ENL genes subset. **(F)** Heatmap representing the UTR enrichment of ENL; **(G)** to RR and;**(H)** to WR.

Functional enrichment analysis revealed a convergence of biological signals across clinical forms, except in the rSm29 vs *M. leprae* comparison in ENL clinical form. Among the DB categories, positive regulation was linked to processes such as organismal death, motor dysfunction, cell degeneration, bleeding, and movement disorders. In contrast, most enriched processes showed negative regulation, including body size, cytoplasm and cytoskeleton organization, formation of cellular protrusions, cell migration, microtubule dynamics, and overall cell movement. In CP module, the main signals included negative regulation of neutrophil degranulation, osteoclast roles in rheumatoid arthritis, *IL-15* and *IL-10* signaling, neuroinflammation signaling, and the positive regulation of *IL-12* signaling and production in macrophages pathway and positive regulation of *IL-12* signaling and production in macrophages (**Figures 4A, B**).

We also identified a distinct RR/WR-specific transcriptional signature comprising 143 genes under rSm29 stimulation. Enrichment analyses for DB pointed to negative regulation of apoptosis, RNA transcription, endocytosis, epithelial tissue necrosis, cell migration, and T lymphocyte proliferation. Similarly, CP analysis revealed negative regulation of *NF-κB* signaling, oxytocin signaling, and pathways involving chondrocytes, macrophages, fibroblasts, and endothelial cells in rheumatoid arthritis, as well as *IL-17* and *IL-6* signaling (**Figures 4C, D**).

Finally, we identified a unique transcriptomic signature of 23 genes exclusive to ENL under rSm29 stimulation. Here, enriched DB categories showed negative regulation of angiogenesis, cell migration, and invasion (**Figure 4E**). However, no CP reached the z-score threshold to predict activation or inhibition (**Supplementary Figure 3**).

To further explore the regulatory mechanisms driving these transcriptional responses, we conducted an UTR analysis for each clinical form. In ENL under rSm29 stimulation, all major UTRs were predicted as negatively regulated, including *TNF, IL1B, CCL5, CSF2, TLR4*, and *TLR* (**Figure 4F**). In the RR group, rSm29 stimulation compared to *M. leprae* led to strong predicted inhibition of key pro-inflammatory regulators, such as *IFNG, NONO, TNF, STAT1, CSF2* and *IFN-α*, suggesting an anti-inflammatory effect of rSm29 (**Figure 4G**). In contrast, WR patients showed a positive upstream regulatory profile under rSm29 stimulation, with predicted activation of *IL1B, NFKB, CD3*, and *TNF*, indicative of a more pro-inflammatory response (**Figure 4H**).

### 3.7 Immune composition across groups and stimuli

**Figure 5:**
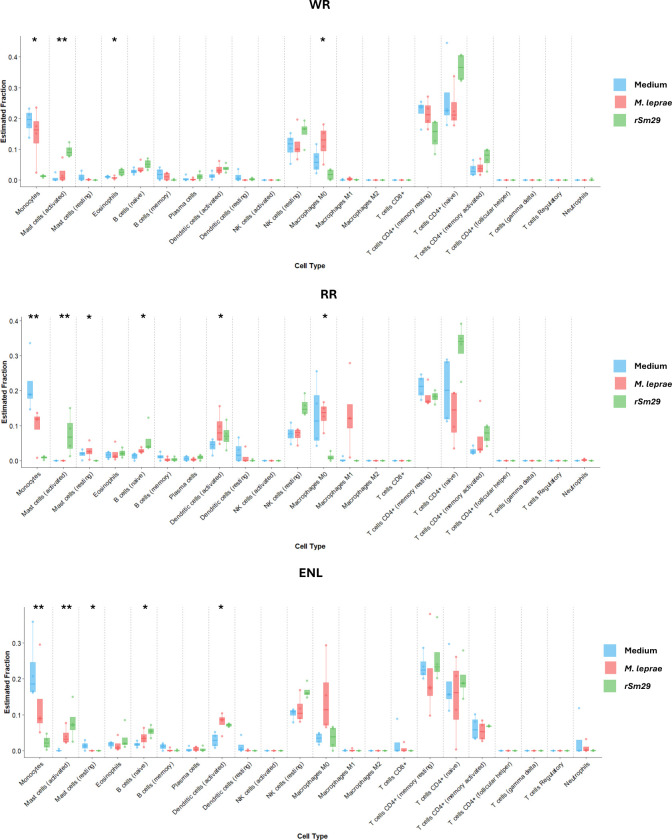
Estimated immune fraction composition in clinical forms under three stimulation conditions. Boxplots represent the estimated fraction of each cell type (relative values ​​between 0 and 1) obtained by deconvolution from gene expression data normalized by DESeq2. Within each clinical group, three stimulation conditions are shown: unstimulated medium (blue violins; dark−blue points), *M. Leprae* (pink violins; dark−pink points), and rSm29 (green violins; dark−green points). In each boxplot, the central line indicates the median; the box limits correspond to the first and third quartiles; the points represent individual samples. Asterisks indicate the level of statistical significance between conditions for each cell type (*p < 0.05; **p ≤ 0.01), assessed by Kruskal–Wallis test. The cell types evaluated are shown on the X-axis. **(A)** for WR, **(B)** for RR, and **(C)** for ENL.

In the WR, activated mast cells were markedly enriched following rSm29 stimulation (p ≤ 0.01), with their fractional abundance higher than under *M. leprae* stimulation or medium. Conversely, monocytes and M0 macrophages were significantly reduced after rSm29 exposure. Eosinophil fractions also differed across stimuli, and although not statistically significant, naive and memory-activated CD4+ T cells trended upward with rSm29 (**Figure 5A**).

In RR, changes were more pronounced and cell-type specific. Monocytes and activated mast cells exhibited the strongest differences (p ≤ 0.01) but in opposite directions: monocyte fractions were highest in medium (and secondarily with *M. leprae*) and were reduced under rSm29, whereas activated mast cells were predominantly detected after rSm29 and were negligible under the other conditions. Resting mast cell fractions show counts in medium and *M. leprae*. M0 macrophages showed higher fractions in medium and *M. leprae* than in rSm29. Activated dendritic cells varied significantly across stimuli. As in WR, naive and memory-activated CD4+ T cells tended to increase with rSm29 without reaching significance (**Figure 5B**).

In ENL the pattern closely mirrored RR. Monocytes decreased significantly with rSm29 relative to the other stimuli (p ≤ 0.01) and activated mast cells increased strongly with rSm29 (p ≤ 0.01). M0 macrophages were reduced, and naive B cells were elevated under rSm29. Activated dendritic cells showed stimulus-dependent differences, with the largest response to *M. leprae*. CD4+ T cell subsets in ENL were comparatively stable across stimuli (**Figure 5C**).

## 4 Discussion

The marked, stimulus−dependent cytokine and chemokine signatures measured by ELISA motivated our transcriptomic investigation: antigenic stimulation with rSm29 produced consistent and significant increases in key mediators (notably *TNF*, IL−6 and IL−10, with IL−5 and CXCL9 showing stimulus− and group−specific patterns), and these protein−level differences were most pronounced in reactional patients, particularly those with RR. The Sm29 is correlated with *IL-10 induction*, as a characteristic attributed to its modulating function (6–8). Our data is consistent to this and underpinned by the transcriptome, where up regulation of *IL-10* gene is highlighted in RR. An antigenic stimulus *per se* may evoke promptly inflammatory cytokines such as *TNF* and *IL-6*, as they can be already contained in cellular vesicles by other stimuli. However, at the transcriptomic level, the Sm29 modulated both genes. This highlights that the antigen has the potential to induce this modulatory response but also, that subsequent pos-transcriptional mechanisms also influence its production. Regarding *IL-5*, the higher levels observed in patients without reactions may be due to the fact that 3/4 of these individuals are in the lepromatous pole of the disease, in which a more Th2-type response naturally predominates. Anyway, because ELISA results indicated a coherent antigen−specific immune activation that could not be fully explained at the protein level alone, we performed RNA−seq on stimulated PBMCs to map upstream regulators, cellular programs and pathway networks underlying these responses, which will be the focus of this discussion from now on.

Transcriptomic profiling revealed stimulus− and group−specific programs. PCA separated rSm29−stimulated samples from others in WR and RR, while ENL samples differed more subtly along PC2. Likewise, differential gene expression analysis revealed pronounced transcriptional changes in both RR and ENL, while the WR group showed no significant DEGs between *M. leprae* stimulation and the medium control. These results motivated further network and deconvolution analyses to identify pathways and cellular compositions specific to each clinical form.

Deconvolution analysis revealed stimulus- and clinical-form-specific shifts in inferred immune composition. Overall, *M. leprae* stimulation tended to preserve or amplify myeloid, pro-inflammatory signatures: higher estimated fractions of monocytes and macrophage-related phenotypes accompanied by dendritic cell activation, consistent with canonical Th1 polarization (IFN-γ/TNF-α) and recruitment of inflammatory effectors. By contrast, rSm29 produced a reproducible immunomodulatory pattern: relative depletion of monocyte and M0/macrophage signatures, indicative of attenuated innate/Th1 signaling, accompanied by enrichment of elements linked to type-2 and humoral immunity, notably activated mast cells, eosinophils and increased naive B cells, with trends toward activation of CD4+ naive and memory subsets.

Stratified analyses showed WR to be milder and heterogeneous, with a pronounced expansion of activated mast cells under rSm29 (p ≤ 0.01) and reduction of monocyte/M0 signatures; RR exhibited more marked remodeling, with monocyte/M0 signatures highest in medium or after *M. leprae* and reduced with rSm29, while activated mast cells and lymphoid features increased under rSm29; ENL paralleled RR, showing decreased monocyte/M0 estimates and increased activated mast cell and B-cell signals with rSm29, whereas *M. leprae* retained stronger dendritic/myeloid/chemotactic signatures.

Integration with ELISA cytokine profiles supports a coherent functional interpretation: antigen-driven increases in *TNF* and *IL-6* in reactional patients and concurrent *IL-10* induction, shifts the immune landscape away from classical macrophage-centric Th1 programs toward type-2/humoral and regulatory programs (*IL-4/IL-5/IL-10*–associated biology), with the effect size dependent on baseline inflammatory tone. Important caveats apply the deconvolution yields relative proportions based on a reference signature matrix and transcriptional states, so apparent decreases may reflect true numeric loss, differentiation to transcriptionally distinct states, or relative expansion of other compartments, and algorithmic misclassification is possible. In summary, rSm29 imposes a consistent immunomodulatory program across clinical forms, attenuating innate, macrophage-centric (Th1) signatures while enhancing type-2/humoral features, warranting mechanistic follow-up and evaluation as an immunomodulatory candidate.

These multi−layered data therefore indicate a coherent, context−dependent effect of rSm29: at the protein and inferred cell−type level rSm29 skews responses away from macrophage−centric Th1 biology, yet the transcriptional response it elicits is qualitatively distinct in RR versus ENL. Specifically, genes highly upregulated by rSm29 in RR were among the least expressed in ENL, integrating ELISA and PCA results show that the same antigen triggers qualitatively different transcriptional programs depending on the baseline immune context. In RR, characterized by a strong Th1/IFN−driven cellular response, rSm29 unmasks and amplifies a lymphoid module with regulatory components (consistent with protein profiles and clear PCA separation). In ENL, characterized by systemic inflammation, that lymphoid/regulatory module is not engaged or is suppressed in favor of chemotactic pathways. This pattern corroborates classical descriptions and indicates qualitative, not merely quantitative, differences between RR and ENL.

Although a shared signature of 235 modulated genes was identified across all groups, the expression levels diverged markedly between REAC. This discrepancy suggests that, while a common transcriptional response exists, the underlying immune activation pathways differ drastically according to the clinical context, directing unique immunological mechanisms in each patient group. In the 235 genes signature, IPA analyses demonstrating more pronounced downregulation of key pathways in RR, emphasizing the modulating characteristic of the rSm29. The disease pathways are related to a profile involving neuroinflammation (neuroinflammation signaling pathway, Parkinson’s signaling pathway) and immune cell response in rheumatoid arthritis (RA). This information in turn is interesting, since there are data associating leprosy to a profile of genes validated for autoimmune diseases (20). In addition, we highlight a modulation in functional process related to macrophage degranulation, phagocyte formation and S100 family signaling pathway. Previous data show that depending on the antimicrobial pathway, macrophages can become more or less phagocytic, with phagocytic activity being linked to the most disseminated and multibacillary form of the disease (21). Also, *PGL-1* induces nitric oxide synthase in infected macrophages, inducing demyelination by contact with axons and initiating nerve damage in leprosy (22). In addition, members of the S100 protein family act as regulators of a variety of cellular processes, usually by binding Ca2+, but also Zn2+ and Cu2+ and acting as a marker of neurotoxicity. Immunohistochemical expression or its serum levels have been determined in several clinical disorders, including leprosy (23, 24).

The unique 143-gene signature in the RR/WR group and the exclusive 23-gene module in ENL underline the heterogeneity in response to rSm29 across clinical forms. In RR, the prominent downregulation of pathways related to inflammatory cell activation — like mechanisms observed in rheumatoid arthritis — contrasts with a milder response in ENL, highlighting the antigen’s context-dependent effects. In RR, the induction of the regulatory cytokine *IL-10*, reaffirming previous findings in CL (6, 25) and asthma (26) underscores the anti-inflammatory action of this antigen. Concurrently, we observed modulation of key mediators, including members from the *IL1B* and *TNF* families and *IFNG*, suggesting a mechanism that balances pro- and anti-inflammatory signals, all well-documented contributors to the immunopathogenesis of leprosy (27–29). IPA analyses showed that the UTR for the positive modulation of *IL10* were the *CSF2* and *IFN-α* genes. *CSF2*, also known as granulocyte macrophage-colony stimulating factor, GM-CSF, is a cytokine that stimulates cells of the hematopoietic lineage and induces protective immunity, mainly by stimulating the recruitment, maturation, and functioning of dendritic cells (30). IFN-α is a member of the type I interferon family that is produced in response antigenic stimulus as a part of the innate immune response. Previous data from our own group show a signature for RR in whole blood, comprising genes including type I *IFN* components, autophagy and Toll like receptors (31).

Together, the regulation of *CSF2* and *IFN-α* by rSm29 impacted a series of key genes, may have great biological relevance, since continuous binding of PAMPs and DAMPs to TLRs provides the necessary trigger to maintain the inflammatory process and stimulate innate mechanisms with autophagic activities, having type I *IFNs* ahead of the process. In addition to *IL-10*, Sm29 also increased *PTGS1*, and this network modulates processes such as cell migration, proliferation, angiogenesis, as well as cellular degeneration (32). Overall, in RR, the most notable canonical pathways related to the Sm29 antigen effect are the downregulation of rheumatoid arthritis-related cells (chondrocytes, macrophages, fibroblasts, endothelial cells, osteoclasts, and osteoblasts), which is in accordance to the AR regulation profile signature mentioned a little above, as well as cytokine signaling (we emphasize *IL-17* and *IL-6*), as well as *NF-kB* signaling.

Likewise, samples from WR patients showed the same profile when stimulated by rSm29. This similarity may indicate a profile of patients who might develop RR *a posteriori*, however, our sample is small, and we would need to confirm this finding following a larger number of participants to validate this hypothesis.

Finally, we identified a 23-gene signature unique to ENL, although this smaller module did not yield canonical pathways reaching the z−score threshold for activation or inhibition network analysis highlighted modulation of the *IL1B* and *FN1* genes which in turn regulate chemokines and cytokines such as *IL-6, CCL2* and *CXCL8 (IL-8).* The role of these cytokines in leprosy, especially in leprosy reactions, has already been well described in the literature. For example, *CCL2* in leprosy had been reported in earlier studies (33, 34); *IL-6* was previously related to reactions and higher levels of *IL-6* was also associated with progression from MB disease to ENL (34, 35); whereas *CXCL-8* is induced by *TNF* and detected in skin biopsies from lesions across the leprosy spectrum and also in reactional patients. This set should therefore be interpreted as preliminary, offering leads that may inform future investigations into systemic responses in ENL.

Currently, leprosy reactions are primarily managed with variable doses of corticosteroids, and in the case of ENL, treatment options include thalidomide (100 to 400 mg/day) or corticosteroids (with an initial dose of 0.5 to 1 mg/kg body weight/day) administered over prolonged periods (36). However, thalidomide is highly teratogenic, and its use is limited in women of childbearing age and extended corticosteroid therapy can lead to significant metabolic side effects affecting ophthalmological, cardiovascular and musculoskeletal systems. Alternatives, such as combinations of clofazimine with prednisone or pentoxifylline, have been explored but do not match the therapeutic efficacy of thalidomide. Moreover, given that the pathophysiology of ENL involves the deposition of antigen-antibody complexes and systemic elevated *TNF* and other inflammatory cytokines, newer immunobiological agents such as etanercept and infliximab as well as cyclosphopahmide pulse therapy have been reported with good results (37). This reliance on corticosteroids and thalidomide, drugs associated with significant adverse effects, underscores the urgent need for safer interventions.

Our findings suggest that rSm29 is associated with modulation of key inflammatory pathways, raising the prospect that targeting these pathways could form the basis for developing new, less toxic therapeutic strategies, particularly in ENL where excessive *TNF* production is a central driver of pathology. In this context, the identification of antigens, peptides, or other biologically active products with the potential to treat leprosy reactions is highly relevant for disease management and may help prevent disabilities resulting from neural damage.

The study design, which used non-reactional patients as the reference group, provided a clinically meaningful baseline for comparisons across the spectrum of leprosy reactions. This approach emphasizes disease-contextual differences rather than contrasts with healthy individuals, which we consider a strength. At the same time, the absence of a healthy control group limits the ability to fully distinguish disease-specific effects from general immune responses, and future studies incorporating healthy controls will be valuable to refine these distinctions.

Although encouraging, our results should be interpreted as exploratory due to the relatively small sample size (n=4 per group), highlighting the need for validation in larger, more diverse cohorts … The regulatory networks identified here, including CSF2/IL10 and FN1/IL1B/chemokine axes, remain putative and warrant further substantiation through functional approaches. Taken together, these results provide initial insights into the immune modulation associated with leprosy reactions and lay the groundwork for future studies that can expand and confirm these observations. Additionally, the cross-sectional design and reliance solely on transcriptomic data provide only a snapshot of immune responses, without capturing dynamic changes over time or under varying treatment conditions. Future investigations that incorporate longitudinal analyses and functional assays will be essential to validate the predicted regulatory networks and further elucidate the mechanisms by which rSm29 modulates immune responses.

In summary, our findings demonstrate that rSm29 triggers distinct and context-dependent transcriptomic responses across the clinical forms of leprosy. The complex modulation of immune pathways highlights the potential of rSm29 to serve as a basis for developing novel, less toxic therapeutic strategies.

## 5 Conclusion

This study demonstrates the capacity of rSm29 to modulate the intense inflammatory process of leprosy reactions, identifying an immunobiological product that could advance in research for future use in specific clinical conditions, where an alternative to the recommended treatments would be sought. Advances in knowledge in this area have important consequences for preventing disabilities and reducing morbidity associated with the use of current therapies, especially in cases of refractoriness.

## Data Availability

The original contributions presented in the study are publicly available. This data can be found here: BioProject accession number PRJNA1463881.

## Supplementary material

Supplementary Figure 1 Volcano plots.

Supplementary Figure 2 Upset.

Supplementary Figure 3 CP of 23 genes subset for ENL.

Supplementary Table 1 DEGs.
