# Supplementary figures and images for: The Sm29 antigen differentially shapes transcriptomic and regulatory landscapes across reactional forms of leprosy
Source: Front Immunol. 2026 Jun 1;17:1844112. doi: 10.3389/fimmu.2026.1844112 (PMC13265286; doi:10.3389/fimmu.2026.1844112)

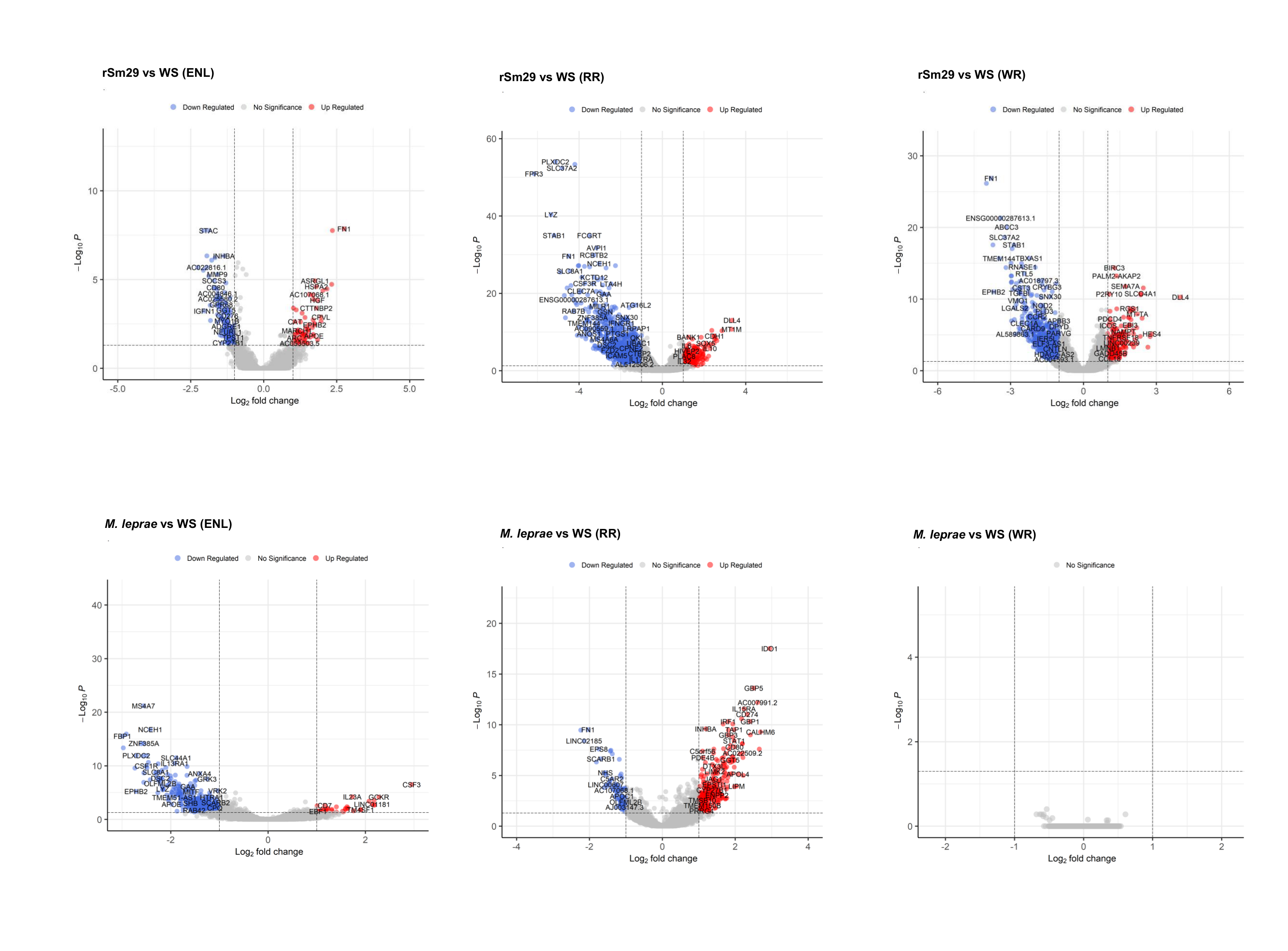

Supplement: Supplementary Figure 1 — Volcano plots. [file Image1.tiff]

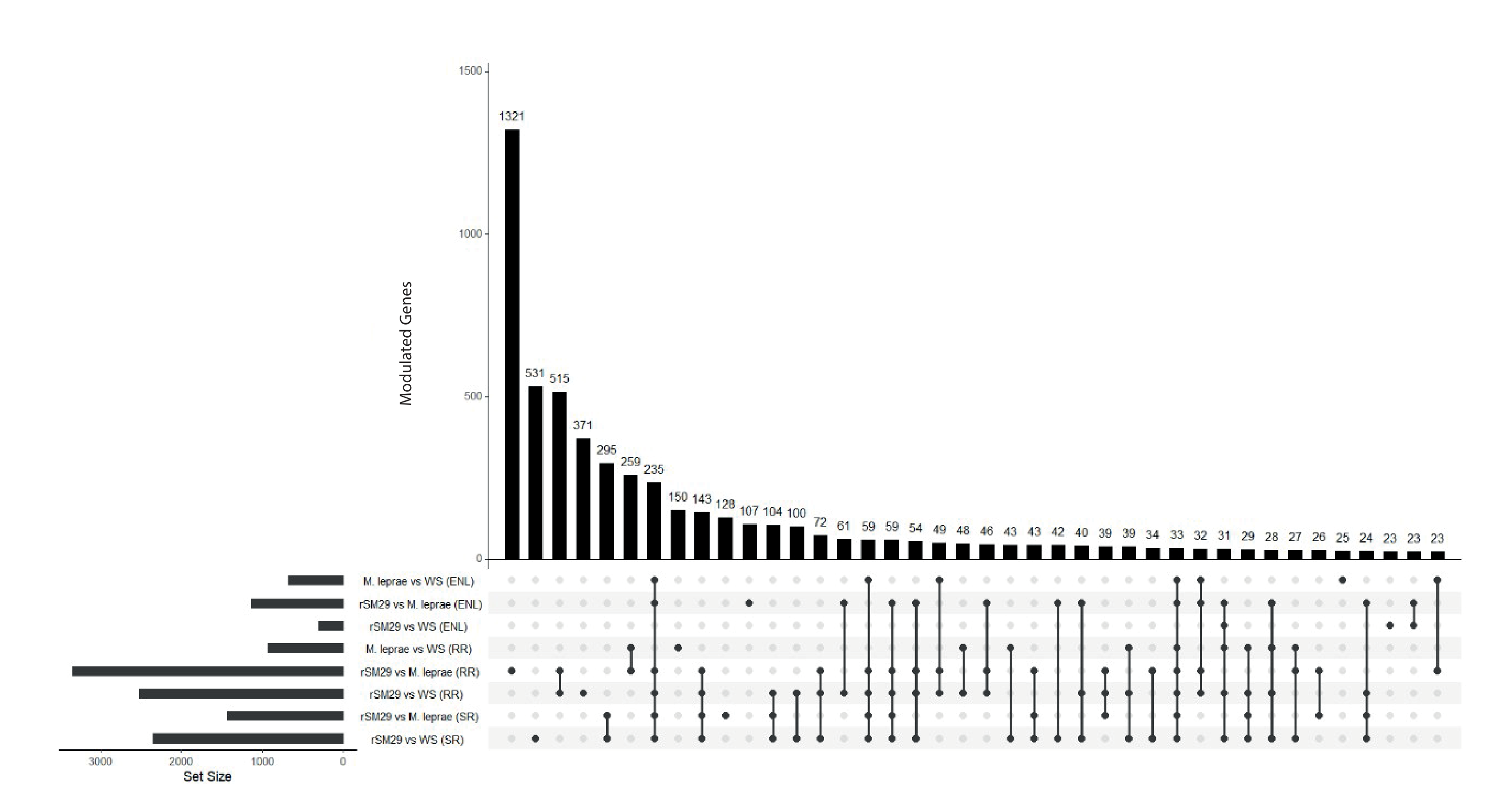

Supplement: Supplementary Figure 2 — Upset. [file Image2.tiff]

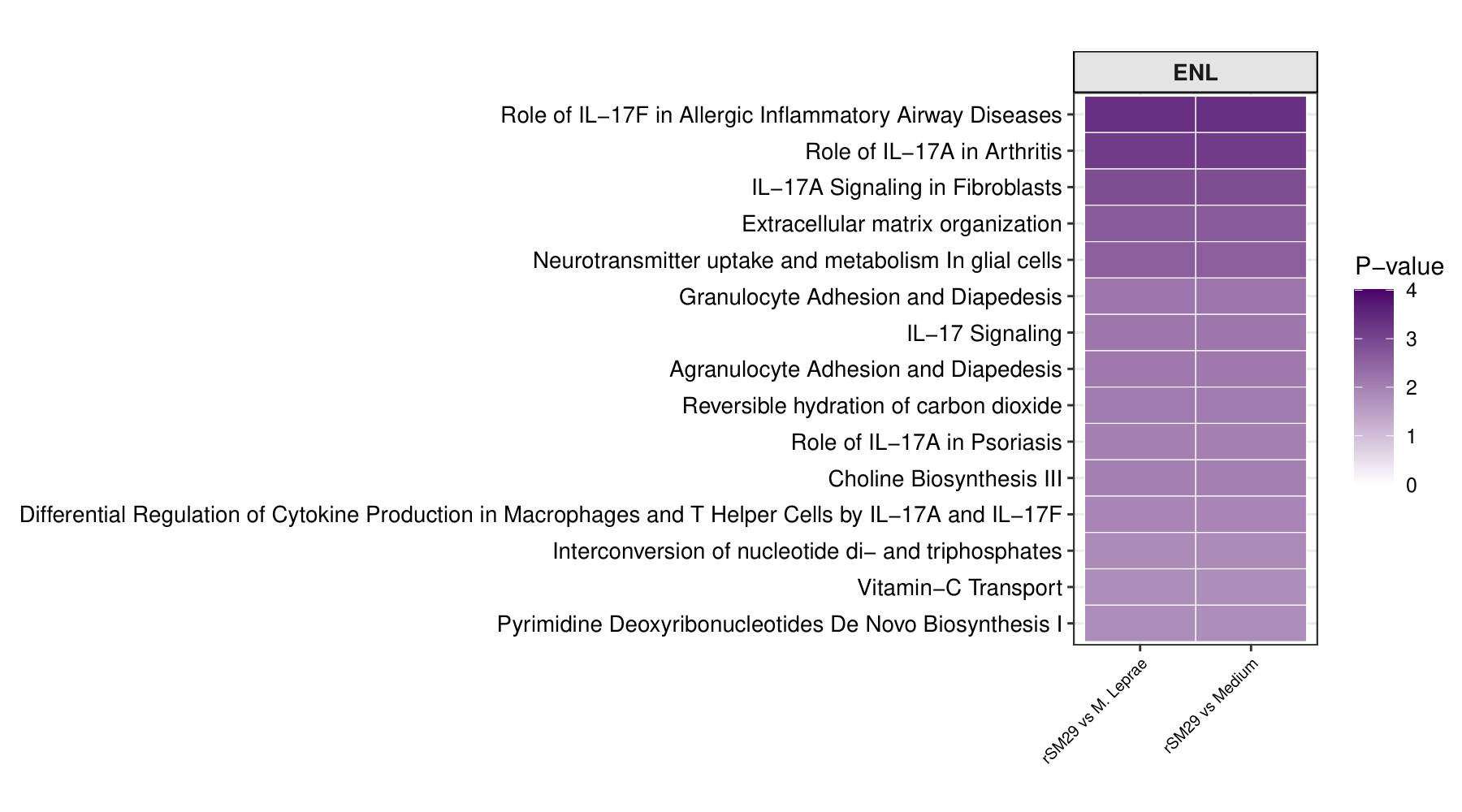

Supplement: Supplementary Figure 3 — CP of 23 genes subset for ENL. [file Image3.tiff]
